# Evolution of self-care in patients with heart failure at the first outpatient return and three months after hospital discharge*

**DOI:** 10.1590/1518-8345.4364.3440

**Published:** 2021-07-19

**Authors:** Debora Cristine Previde Teixeira da Cunha, Lidia Aparecida Rossi, Carina Aparecida Marosti Dessote, Fabiana Bolela, Rosana Aparecida Spadoti Dantas

**Affiliations:** 1 Hospital do Coração, São Paulo, SP, Brazil.; 2 Scholarship holder at the Coordenação de Aperfeiçoamento de Pessoal de Nível Superior (CAPES), Brazil.; 3 Universidade de São Paulo, Escola de Enfermagem de Ribeirão Preto, PAHO/WHO Collaborating Centre for Nursing Research Development, Ribeirão Preto, SP, Brazil.

**Keywords:** Heart Failure, Self Care, Nursing Care, Ambulatory Care, Health Education, Cardiology, Insuficiência Cardíaca, Autocuidado, Cuidados de Enfermagem, Assistência Ambulatorial, Educação em Saúde, Cardiologia, Insuficiencia Cardíaca, Autocuidado, Atención de Enfermería, Atención Ambulatoria, Educación en Salud, Cardiología

## Abstract

**Objective:**

to analyze the evolution of self-care in hospitalized patients with decompensated heart failure, between the first return after hospital discharge (T0) and three months after this assessment (T1).

**Method:**

an observational, analytical and longitudinal study carried out in the cardiology outpatient clinics of two public hospitals in Ribeirão Preto, São Paulo. The sociodemographic and clinical data were collected through interviews and consultation of medical records. Self-care was assessed using the Brazilian version of the Self-Care of Heart Failure Index-SCHFI instrument. The data were analyzed by means of the Student’s t test and paired distribution (McNemar) with a significance level of 0.05.

**Results:**

we verified an increase in the mean scores of the three subscales of SCHFI (Maintenance, Management and Confidence), when comparing the values of T0 and T1, these differences being statistically significant (p<0.001). When comparing the positive changes in self-care actions over these months, we found statistically significant changes in the Maintenance (6 out of 10 items), Management (5 out of 6 items) and Confidence (4 out of 6 items) subscales.

**Conclusion:**

self-care for heart failure improved in the period between the first return after discharge and the end of three months of follow-up. Further studies are needed to verify the variables associated with improved self-care after hospitalization.

## Abstract (pt)

**Objetivo:**

analisar a evolução do autocuidado de pacientes internados com insuficiência cardíaca descompensada, entre o primeiro retorno após alta hospitalar (T0) e três meses após essa avaliação (T1).

**Método:**

estudo observacional, analítico e longitudinal realizado nos ambulatórios de cardiologia de dois hospitais públicos de Ribeirão Preto, São Paulo. Os dados sociodemográficos e clínicos foram coletados por entrevistas e consulta aos prontuários. O autocuidado foi avaliado pela versão brasileira do instrumento *Self-Care of Heart Failure Index*-SCHFI. Os dados foram analisados pelos testes de t de Student e distribuição pareada (McNemar) com nível de significância de 0,05.

**Resultados:**

constatamos aumento nas médias dos escores das três subescalas do SCHFI (Manutenção, Manejo e Confiança), quando comparados os valores de T0 e T1, sendo essas diferenças estatisticamente significantes (p<0,001). Ao compararmos as mudanças positivas nas ações de autocuidado ao longo desses meses, encontramos mudanças estatisticamente significantes nas subescalas Manutenção (6 dos 10 itens), Manejo (5 de 6 itens) e Confiança (4 de 6 itens).

**Conclusão:**

o autocuidado da insuficiência cardíaca melhorou no período entre o primeiro retorno após a alta e o final de três meses de acompanhamento. Outros estudos são necessários para verificar as variáveis associadas à melhora do autocuidado após a internação.

## Abstract (es)

**Objetivo:**

analizar la evolución del autocuidado en pacientes hospitalizados con insuficiencia cardíaca descompensada, entre el primer retorno tras el alta hospitalaria (T0) y tres meses después de esa evaluación (T1).

**Método:**

estudio observacional, analítico y longitudinal realizado en las consultas externas de cardiología de dos hospitales públicos de Ribeirão Preto, São Paulo. Se recogieron datos sociodemográficos y clínicos mediante entrevistas y consulta de historias clínicas. El autocuidado se evaluó mediante la versión brasileña del instrumento *Self-Care of Heart Failure Index*-SCHFI. Los datos se analizaron mediante la prueba t de Student y distribución pareada (McNemar) con un nivel de significancia de 0,05.

**Resultados:**

encontramos un aumento en las puntuaciones medias de las tres subescalas del SCHFI (Mantenimiento, Gestión y Confianza), al comparar los valores de T0 y T1, siendo estas diferencias estadísticamente significativas (p <0,001). Al comparar los cambios positivos en las acciones de autocuidado durante estos meses, encontramos cambios estadísticamente significativos en las subescalas Mantenimiento (6 de 10 ítems), Gestión (5 de 6 ítems) y Confianza (4 de 6 ítems).

**Conclusión:**

el autocuidado de la insuficiencia cardíaca mejoró en el período comprendido entre el primer retorno tras el alta y el último a los tres meses de seguimiento. Se necesitan más estudios para verificar las variables asociadas con la mejora del autocuidado después de la hospitalización.

## Introduction

With the increase in the number of older adults in the world population, new chronic diseases started to appear in society, among which we can mention heart failure (HF)^1^^-^ 2. In Brazil, the diseases affecting the circulatory system were responsible for 1,151,522 deaths in 2018, with 200,760 deaths caused by HF^3^. HF is a complex and multi-factorial chronic syndrome and can occur due to cardiac, structural and functional abnormalities resulting from several other diseases, mainly those of cardiovascular origin, resulting in reduced cardiac output. Its treatment is complex and involves measures of a pharmacological and non-pharmacological nature^4^^-^ 6. Failure to adhere to treatment can lead to episodes of decompensation, characterized by the onset, either sudden or gradual, of typical symptoms such as dyspnea, edema in the lower limbs, fatigue, and also by the presence of jugular stasis and lung crackles^6^^-^ 7. Non-pharmacological treatment is usually carried out in the home environment, as it involves lifestyle-related measures such as daily weight control, reduced sodium, fluid and alcohol consumption, increased physical activity, and immunization, in addition to the use of the medications prescribed^5^^-^ 6. The complexity of the clinical condition and the deficiency of self-care can justify the high rates of hospital readmissions due to the decompensation of HF^1^^-^ 2 ^,^ 4 ^,^ 7.

Although the focus of the self-care concept is predominantly linked to health promotion and disease prevention^8^, self-care actions are also developed by people in the face of the appearance of signs and symptoms of cardiovascular diseases, for example^9^. In the present study, self-care was investigated according to the definition of North American researchers who consider it as a decision-making process that involves behaviors aimed at maintaining physiological stability, monitoring and managing the symptoms of heart failure. It is an active and planned process that permeates the following stages: recognition of a symptom (for example, gaining body weight without changing the eating pattern and onset of dyspnea), assessing the change in health status, the decision to implement a treatment strategy (for example, taking an extra dose of diuretic, as prescribed) and evaluating the treatment/action performed (weight loss and improved breathing pattern)^10^^-^ 11. Based on this definition, the authors developed an instrument for assessing self-care in HF that assesses three dimensions of self-care: Maintenance (it involves monitoring, adherence to treatment, and recognizing the signs and symptoms of decompensation); Management (it covers the assessment of signs and symptoms by the patient, their attitudes towards them and the reevaluation seeking to improve these signs and symptoms of HF) and Confidence (it encompasses both maintenance and management of HF). It is expected that, when obtaining positive results of adherence to treatments, both pharmacological and non-pharmacological, the patient will feel more confident in the management of HF^10^.

The patients’ knowledge about their health condition, self-care and adherence to therapy reduces the morbidity and mortality caused by the disease. Teaching strategies focused on pharmacological measures, such as adherence to treatment and monitoring of symptoms, and non-pharmacological measures, such as changes in diet, physical activity and daily weight have been shown to be effective in improving the quality of life and prognosis of these patients^6^^,^ 12. Nursing is one of the professions of the health team that monitors the treatment of patients with HF, focusing on strategies and activities to improve self-care^13^^-^ 14. In Brazil, the investigation of the self-care of patients with HF has occurred mainly through cross-sectional studies. Thus, our study is important because it evaluated how self-care evolves after hospitalization due to decompensation of the syndrome.

The present study aimed to analyze the evolution of self-care in patients who have been hospitalized with decompensated heart failure, in the period between the first return after hospital discharge from the last hospitalization and the third month after the first contact.

## Method

An observational and longitudinal study carried out in the cardiology outpatient clinics of two public hospitals in the inland of São Paulo. This study was approved by the Ethics Committee of the Ribeirão Preto School of Nursing under CAAE number 57335816.0.0000.5393. The participants and the researcher signed the Free and Informed Consent Form (FICF).

The population of interest consisted of hospitalized patients with decompensated HF. Potential participants were identified by a daily active search in the inpatient units and during the first outpatient return visit, after discharge from the last hospitalization due to decompensated syndrome. At that time, the eligibility criteria were verified. The potential participants were invited to the survey, which involved three months of follow-up. The sample was consecutive and non-probabilistic, with the recruitment period between November 2016 and June 2018. The inclusion criteria were adult patients (18 years old or older), of both genders, hospitalized with the diagnosis of decompensated HF reported in their medical records, and who returned for the first outpatient assessment. Those who had the following characteristics were excluded: presence of hearing loss; diagnosis of mental disorder described in the medical record; and those who did not have clinical conditions to participate in the interview (presence of signs and symptoms such as fatigue and/or dyspnea). In the second evaluation, participants were excluded who, during the three-month follow-up, required surgical interventions, installation of a permanent pacemaker or implantable defibrillator cardioverter.

Data was obtained through individual interviews and consultations in the participants’ medical records. The interviews were conducted by one of the researchers (DCPC), at the two moments of the investigation: first outpatient return after hospital discharge from the last hospitalization for decompensated HF (T0) and three months after the first evaluation (T1). Sociodemographic (gender, age, income, marital status and presence of caregiver) and clinical data [medications used, comorbidities, etiology of HF, left ventricular ejection fraction (LVEF) obtained in the echocardiographic examination of the participant and that later was categorized as preserved (≥50%), intermediate (40-49%) and reduced (≤40%)]^6^ were collected, as well as those related to HF decompensation. At the time of hospitalization due to decompensation, it is important to consider the clinical-hemodynamic profile, using the congestion and perfusion parameters of the organs^15^, to support the therapeutic decision. Upon being hospitalized for decompensation, the patients will be characterized regarding the clinical profile of HF, according to the following order: characteristic of tissue perfusion (positive/hot or negative/cold) and level of congestion (positive/wet or negative/dry). After this evaluation, they will be classified between profiles A, B, C or L. The most common profile is B (hot and humid), in which perfusion is adequate, although congestion is present^4^^,^ 15. To assess the severity of the symptoms and the tolerance to physical activities of the patients, in the two outpatient assessments, we used the self-reported functional class^16^, adapted from the system proposed by the New York Heart Association^17^.

For the assessment of HF self-care, we used the Self Care of Heart Failure Index v. 6.2 (SCHFI)^14^ in its version adapted to Portuguese^18^. Permission to use the scale in the Brazilian version was requested and granted by the authors. This instrument assesses self-care in three subscales: Maintenance (10 items), Management (6 items) and Confidence (6 items). In the Maintenance domain, the answers for each item vary from “never/rarely” (value 1) to “always/daily” (value 4); for the Management domain, they range from “unlikely” (value 1) to “very likely” (value 4); and, for the Confidence domain, from “not confident” (value 1) to “extremely confident” (value 4). The values of the sum of the scores in each domain are transformed into a standardized scale from 0 to 100. Higher values indicate better self-care according to each subscale^14^. In the validation for Portuguese, the hospitalized consistency evaluated by Cronbach’s alpha was 0.43 for Maintenance, 0.76 for Management and 0.94 for Confidence^18^.

In this study, we used the measures obtained in the domains as quantitative variables and, in addition, the self-care measures according to the categories so that the evolution of the participants was observed, comparing the alternatives chosen at T0 with those indicated at T1. Our categorization proposal was carried out according to the degree of similarity between the response scales, for example, in the Maintenance subscale, the responses ranged from 1 to 4 and were grouped into two categories (example: grouping “Never or Rarely/Sometimes” or “Frequently/Always or Daily”). When the subscale had more than four answer options, a new category was created (example: item 1 of the Management subscale has six answer categories, which were grouped into four categories: “I did not have such symptoms”, “I did not recognize”, “it took me a short/long time to recognize” and “I recognized quickly/immediately”). We categorized the answer scales respecting the similarity pattern of the alternatives in order not to compromise the interpretation of the results.

The data were processed and analyzed using the Statistical Package for the Social Sciences (IBM^®^ SPSS) 25.0 for Windows and the R Program, version 3.5.3. Descriptive analyses of simple frequency were carried out for the categorical variables and analysis of measures of central tendency and of variability for the numerical variables (age, years of study, monthly family income, number of comorbidities). We used the Student’s t test for paired samples and, to analyze the evolution of the participants in the items of the self-care instrument, SCHFI, the McNemar chi-square test. To measure the internal consistency of the SCHFI instrument, we used Cronbach’s alpha coefficient. Values above 0.7 were considered adequate to indicate the reliability of the instrument. The level of significance adopted for the analyses was 0.05.

## Results

During the recruitment period, 245 patients were hospitalized due to HF decompensation; of this total, 186 (76%) patients attended the first outpatient visit and were invited to participate in the study. Eight individuals did not agree to participate and 21 were excluded for presenting one or more of the criteria for exclusion. Thus, 157 patients evaluated at the first return after discharge (T0) participated. On return (T1), which occurred three months after T0, 137 (87%) patients returned to the clinic and completed the study. Of the 20 (13%) patients who did not complete the study, 16 died, two gave up treatment, and two received an indication for heart transplantation in other services.

The sociodemographic and clinical variables of the 157 participants included in the study are described in Table 1.

**Table 1:** Comparison of the sociodemographic and clinical characterization of the 157 participants at the first outpatient return after hospital discharge (T0), according to the completion of the study three months after the first assessment (T1). Ribeirão Preto, SP, Brazil, 2016-2018

Sociodemographic characteristics	Finished (n=137) %(n) / M (S.D.)*	Losses/Exits (n=20) %(n) / M (S.D.)*	p-value
Age (years old)	60.3 (11.8)	60.2 (12.7)	0.97^†^
Income (in Reais)	1,640.2 (693.3)	1,883.9 (1,062.1)	0.19^†^
Years of study	5.4 (4.3)	5.7 (4.1)	0.72^†^
Gender			0.24^‡^
Male	51.1 (70)	65.0 (13)	
Marital status			0.81^‡^
Married/Consensual union	62.8 (86)	60.0 (12)	
Presence of caregiver			0.53^§^
Yes	82.5 (113)	90.0 (18)	
Days between discharge and first return	17.4 (15.1)	15.4 (11.2)	0.57^†^
Etiology of HF			0.79^§^
Ischemic	24.8 (34)	20.0 (4)	
Chagasic	21.9 (30)	30.0 (6)	
Valve-related	13.9 (19)	20.0 (4)	
LVEF^||^			0.90^§^
Preserved	17.5 (24)	20.0 (4)	
Intermediate	4.4 (6)	0	
Reduced	78.1 (107)	80.0 (16)	
Decompensation profile			0.61^§^
Profile B	73.0 (100)	65.0 (13)	
Number of comorbidities	3.3 (1.9)	3.4 (1.6)	0.91^†^
Self-reported CF NYHA^¶^			
Class I	11.7 (16)	27.0 (37)	0.70^§^
Class II	50.4 (69)	60.0 (12)	
Class III	27.0 (37)	20.0 (4)	
Class IV	10.9 (15)	15.0 (3)	

* M (S.D.) = Mean (Standard Deviation);

† Student's t test

‡ Chi-square test;

§ Fisher's exact test

|| LVEF = Left Ventricular Ejection Fraction;

¶ CF NYHA = Functional classification of the New York Heart Association

Among the 137 participants who completed the study, the mean time between hospital discharge and the first outpatient return was 17 days (S.D.=14.6) The majority (84; 61%) returned within the first 15 days; 27 (20%) between 15 and 30 days, 25 (18%) between 31 and 60 days and one participant on the 70^th^ day.

We did not find any statistically significant results when we analyzed, in a bivariate way, the measures of the subscales of the self-care instrument with the sociodemographic and clinical variables, shown in Table 1.

The comparisons of the means of the three subscales of SCHFI, assessed during the first outpatient return after hospital discharge (T0) and at three months after the first contact (T1), revealed statistically significant differences with an improvement in the self-care actions over the evaluated period (Table 2).

**Table 2:** Comparison of the mean scores of the 137 participants at the first outpatient return (T0) and three months after (T1) and the values of Cronbach's alpha of the subscales/domains of SCHFI*. Ribeirão Preto, SP, Brazil, 2016-2018

SCHFI* domains	T0 M (S.D.)^†^	T1 M (S.D.)^†^	p-value^‡^	T0 Cronbach's Alpha	T1 Cronbach's Alpha
Maintenance	39.1 (16.4)	56.8 (14.4)	0.001	0.54	0.64
Management	34.0 (27.3)	47.9 (26.5)	0.001	0.78	0.86
Confidence	46.2 (26.4)	58.5 (26.3)	0.001	0.87	0.90

* SCHFI = Self-care of Heart Failure Index;

† M (S.D.) Mean (Standard Deviation);

‡ Student's t test

We analyzed the means of the items of the three subscales, considering that the values could vary from 1 (worst self-care) to 4 (best self-care). In the Maintenance subscale, the means ranged from 1.3 (S.D.=0.9) (Do you request foods with little salt when eating out or visiting someone?) to 3.7 (S.D.=0.8) (Are you assiduous in the consultations with the doctor or nurse?), at T0, and between 1.7 (S.D.=0.9) (Do you practice some physical activity?) and 3.9 (S.D.=0.8) (Are you assiduous in the consultations with the doctor or nurse?), at T1. When analyzing changes, with the participants changing the answer from “Never/Rarely/Sometimes” (at the first return after discharge) to “Frequently/Always/Daily” (three months after), we verified positive and statistically significant changes (p=0.001) in six of the 10 items: Weigh yourself (62.7% of the participants); Try to avoid getting sick? (81.7%); Be assiduous in the consultations (96.3%); Ingest a low-salt diet (75.2%); Request foods with little salt (28.5%) and Use a system to remind you about your medicines (81.5%).

In the Management subscale, the “Contact your doctor or nurse for guidance” item showed higher means, both at T0 (M=2.2; S.D.=1.3) and at T1 (M=2.8; S.D.=1.2). The lowest means were for the “How sure are you that this remedy helped?” item with values of 1.3 (S.D.=1.4) and 1.8 (S.D.=1.3), respectively, at T0 and T1. The participants changed their answers from “unlikely/little probable” to “probable/very likely” in four of the six items: “Reduce the salt in your diet” (60.5%; p=0.001); “Reduce your fluid intake” (60.6%; p=0.001); “Take a further diuretic” (48.9%; p=0.001) and “Contact your doctor or nurse for guidance” (62.7%; p=0.003).

For the Confidence subscale, the “Be free of the HF symptoms” item obtained the lowest means, both at T0 (M=2.1; S.D.=0.9) and at T1 (M=2.6; S.D.=0.9). The “Follow the recommended treatment” item was the one with the best confidence assessment for self-care, with means of 2.9 (S.D.=1) and 3.0 (S.D.=0.9). Over the three months, the participants changed from “Not confident/Not very confident” to “Very confident/Extremely confident” in the answers of the following items: “Be free of the HF symptoms” (56.2%; p=0.001); “Evaluate the importance of your symptoms” (64.2%; p=0.009); “Recognize the symptoms” (64.9%; p=0.003); “Do something that can relieve your symptoms” (63.5%; p=0.005) and “Assess whether a drug works” (61.3%; p=0.001).

## Discussion

The aim of our study was to evaluate the evolution of self-care in patients who were hospitalized due to HF decompensation for three months. The initial assessment occurred at the first outpatient visit because it was important to analyze how they behaved after hospital discharge. The second evaluation took place three months after the first return. In the investigated group, we verified an evolution in the self-care actions over the three months, according to the values obtained by the SCHFI instrument. Self-care centered on the individual with HF was conceptually proposed by American nursing researchers in 2008 and, since then, these authors have analyzed knowledge on the subject and updated the theoretical model proposed, as well as the instrument designed to measure self-care^10^^,^ 19. Self-care actions in HF result from a decision-making process by the patient. In this process, several factors are involved (personal, environmental and clinical) and influence the patients’ daily decisions and, consequently, their self-care actions. Experience, knowledge, skills and values must be considered when determining these actions^19^.

Other research studies carried out in Brazil^20^ or in other countries^18^^,^ 21, on the self-care of patients in outpatient follow-up using SCHFI, showed results for the score mean in the Maintenance subscale similar to those found in our study (M=56.8; S.D.=14.4) as measured three months after hospital discharge: 50.5 (S.D.=15.7)^18^, 55.2^21^, 57.0 (S.D.=14.3)^18^. In our study, we found higher Self-care Maintenance values at three months when compared to the results obtained in the first assessment after discharge (p=0.001), similarly to a study carried out in Italy^21^ which showed that longer experience with the diagnosis is indicative of greater Self-care Maintenance. However, Brazilian authors^20^ found a weak relationship between the time of experience with the disease and Self-care Maintenance. The result for the Self-Care Management subscale, three months after the first outpatient return after discharge, is close to the results obtained by authors who evaluated outpatients in Brazil (47.0; S.D.=28.3^18^; 50.0; S.D.=20.3^20^) and in Italy (53.2)^21^. Similar findings to those obtained in our study for the Confidence subscale were found in cross-sectional studies by other authors: 54.1^21^, 58.0 (S.D.=25.5)^18^^)^ and 58.1 (S.D.=18.2)^20^, 70.0 (S.D.=16.2)^14^. In our study, the Confidence subscale also showed better scores in the assessment at three months. These results differ from those presented by other authors who investigated whether greater experience with the disease would be associated with greater confidence in self-care^20^^-^ 21.

Our results lead us to think about the difficulty in adhering to physical activity for patients with HF, considering that the “Practice physical activity” (p=0.31); “Practice physical exercise for at least thirty minutes” and “Request foods with little salt when eating out” items were the ones that showed the least adherence (choice of the never or rarely/sometimes alternative) for, respectively, 83.9%, 83.2% and 71.6% of the participants. Other studies assessed the detailed score of each item in the Maintenance subscale and our data corroborate these findings, considering the low score in relation to physical activity and the non-request for food with less salt when eating out^20^^-^ 21.

In the Management subscale, we found statistically differences for the “Recognize HF symptoms”; “Reduce the salt in your diet”; “Reduce your fluid intake”; “Take a further diuretic” and “Contact your doctor or nurse for guidance” items. The strategies pointed out as “likely/very likely” to be used by the participants were the items about contacting a professional for guidance (58.5%) and reducing salt in the diet (51.5%). However, 63.5% of the interviewees chose the “I have not tried anything/I am not sure/Uncertain” alternative regarding the use of the resources presented in this subscale in order to reduce the symptoms of HF decompensation. In a study conducted in Italy^21^, 38.4% of the 631 investigated patients recognized the symptoms of HF by choosing the answer “quickly/immediately”. The items in the Management subscale indicated by the Italian patients were the same as those chosen by our participants (51.5% would reduce salt in the diet and 58.5% would seek a health professional for guidance). However, differently from our interviewees, 60.1% said that they were “sure/absolutely sure” that these self-care measures would help them to control the symptoms of HF. A study carried out in Brazil^20^ identified that hiring a professional for guidance (Mean=3.1; S.D.=1.0) and the mean score considering the use of resources to help solving the problem was 1.4 (S.D.=1.6).

In the Confidence subscale, the “Be free of the HF symptoms”; “Recognize the symptoms”; “Do something that can relieve your symptoms” and “Assess whether a drug works” items showed statistically significant differences between the answer groups, changing from “Not confident/A little confident” to “Very confident/Extremely confident”. In our study, in the two assessments, the subscale items with the highest percentage of answers for the “Very confident/Extremely confident” option were “Follow the recommended treatment” (52.6%), followed by “Evaluate the importance of your symptoms” (35%), results that corroborate the findings of other studies^20^^-^ 21. The item with the least confidence on the part of the participants, throughout the study, was “Be free of the HF symptoms”. This item was also the worst rated in another Brazilian study^20^.

The sociodemographic profile of the participants with a predominance of men, older adults, married or in a stable relationship, retired, with low income and low schooling is consistent with the profile observed in other national and international surveys^16^^,^ 20 ^-^ 24. Although we have found no association between the self-care measures and the gender, age, marital status, family income and schooling variables, the personal characteristics are associated with the process of determining self-care actions in HF^19^. Regarding the clinical variables, according to an adaptation of the NYHA classification^17^ by Brazilian authors^16^, the self-reported functional classes II and III were informed by the majority of the participants (77.4%). In the present research, the functional class was self-reported, through reading by the researcher of the alternatives and indicating by the participant the answer that most reflected their situation at that moment. This procedure was adopted based on an international study^25^ that pointed out a discrepancy between the NYHA assessment performed by the physician and that reported by the patient. According to the authors, the health professional often does not consider all the manifestations reported by the patients during the consultation, which can lead to impaired diagnosis and prognosis, with consequences for the indication of pharmacological and non-pharmacological therapy for HF^25^. If we compare these results with those obtained with the evaluation, by the NYHA functional classification, traditionally used by health professionals, we verify that they are similar to the data obtained in studies that evaluated inpatients or outpatients^16^^,^ 20 ^,^ 26 ^-^ 29.

The same is true for the most frequent etiology, which was ischemic (24.8%), followed by chagasic (21.9%) and decompensation profile B (73%)^18^^,^ 30. As in other national and international studies^20^^,^ 22 ^,^ 31 ^-^ 33, we found that most of the participants (78.1%) presented HF with reduced LVEF, which shows the complexity and severity of the patients who are hospitalized and/or being followed-up on an outpatient basis in the hospitals where the study was conducted.

The results of the present research showed the relevance of evaluating patients’ self-care after hospitalization due to the decompensation of the syndrome, considered a stressful event. This study does not allow conclusions about the factors that influenced the behavioral changes in the self-care subscales, but showed that there was a statistically significant increase in the self-care measures between the two assessments.

Health education is one of the main focuses of cardiac rehabilitation Nursing, and the education of patients with HF must be focused on their needs and, at the same time, must involve their family members or caregivers. Educational actions need to be initiated during hospitalization and continue throughout the patient’s outpatient follow-up. The nurse can use several strategies and resources to develop such actions. The acquisition of skills for self-care in HF is a result of this educational process and aims, primarily, to self-control the symptoms of this complex syndrome^12^.

Decision-making for self-care is a complex process for patients with HF. A better understanding of the nature of this process will assist the health team in understanding it, opting for the best way to teach the patient with a view to adopting self-care measures for HF. Understanding the causes and reasons that are commonly related to failure in this decision-making process by the patient can help professionals to develop strategies that help patients and their families^9^. Nursing is the profession that is closest to the patient with HF during the entire hospitalization, assuming the monitoring and follow-up of their evolution until hospital discharge. A well-established therapeutic plan focusing on patient education for self-care that considers the participation of the caregiver/family may lead to an improvement in the quality of life related to the health of these individuals, and to a reduction in the number of hospitalizations due to decompensated HF and, consequently, in hospital costs.

We considered the following limitations of our study: reduced number of patients who completed the study and a long interval of days between discharge from the last hospitalization due to HF decompensation and the first outpatient return. As recommended in the guidelines of the American Heart Association^34^, the number of days for the first outpatient return should take place between 7 to 14 days after discharge; in our study, the mean was 17 days (S.D.=14.6). At the site under study, the large number of people attending outpatient clinics and the lack of follow-up vacancies for patients after discharge contributes to those with better clinical conditions having their returns postponed and scheduled with a longer time interval than those who have greater severity, which may have influenced the results obtained. The assessment of the participants’ knowledge about self-care in HF was not the objective of the present study. The assessment of this aspect could contribute to the analysis of the data obtained, as well as to the understanding of other variables, which have been investigated in studies on this theme, such as resilience, self-efficacy and social support.

## Conclusion

We verified a positive evolution in self-care, with better scores from the participants in relation to HF when comparing the measurements of the three domains of the SCHFI instrument between the first assessment at the first outpatient return after hospital discharge and three months after that return. When comparing the two moments of assessment of the SCHFI items, the results also showed positive changes (better scores) in most self-care behaviors, addressed in the three subscales (Maintenance, Management and Confidence).
